# “*Connect Local*”: protocol for the evaluation of a codesigned whole of community approach to promote social connection in older adults

**DOI:** 10.3389/fpubh.2024.1342562

**Published:** 2024-05-23

**Authors:** R. Ogrin, E. Robinson, K. Rendell, S. Alrababah, D. Fineberg, K. Fiddes, A. Yerolemou, M. H. Lim, L. Engel, J. A. Lowthian

**Affiliations:** ^1^Bolton Clarke Research Institute, Melbourne, VIC, Australia; ^2^Alfred Health, Melbourne, VIC, Australia; ^3^Australian Disease Management Association, Alfred Health, Melbourne, VIC, Australia; ^4^South Eastern Melbourne Public Health Network, Melbourne, VIC, Australia; ^5^Prevention Research Collaboration, Sydney School of Public Health, Faculty of Medicine and Health, The University of Sydney, Sydney, NSW, Australia; ^6^Iverson Health Innovation Research Institute, Swinburne University of Technology, Melbourne, VIC, Australia; ^7^School of Public Health and Preventive Medicine, Monash University, Melbourne, VIC, Australia

**Keywords:** loneliness, social isolation, community, prevention, early intervention, evaluation, older adults, protocol

## Abstract

**Background:**

There is wide acknowledgement in the literature that social connection is protective against loneliness and depression. More robust research, however, is needed to evaluate interventions that promote social connection. This protocol paper outlines the evaluation of a community-wide social connection program, *Connect Local*, in metropolitan Melbourne, Australia to support people 65 years and older to increase access to local community services/activities; and to ascertain impact on social connection, loneliness, depressive symptoms, physical and mental wellbeing, and use of health services.

**Methods:**

A Type 1 Hybrid design, including program effectiveness, cost-effectiveness, and implementation evaluation of the *Connect Local* program, will be undertaken. Eighty-eight participants aged ≥65 years with one or more chronic health condition, who are also either experiencing or at risk of loneliness, social isolation and depressive symptoms will be invited to participate in the evaluation. Outcomes, measured at baseline, 3, 6 and 12 months, include loneliness, social isolation, depressive symptoms, social anxiety, goal attainment, wellbeing, quality of life and health care utilisation. A gender and age matched comparator group of 88 individuals will be recruited from outside the intervention local government area. Impact of the intervention on community service providers in the target region will be evaluated using mixed methods, where triangulation will be used to combine the qualitative and quantitative data using a deductive-simultaneous design. Changes in wellbeing and quality of life of community volunteers will also be measured. All groups will be interviewed to ascertain their experience and perceptions of the program. The economic evaluation will use a Social Return on Investment (SROI) approach, to include outcomes at the individual, community, and system levels. Implementation outcomes will consider Reach, Adoption, Feasibility, Acceptability, Appropriateness, Fidelity, and Sustainability of the intervention.

**Discussion:**

This study will provide a better understanding of the impacts of a community-wide social connection approach in older adults, the community and broader system.

**Clinical trial registration:**

https://www.anzctr.org.au/Trial/Registration/TrialReview.aspx?id=385192; Identifier ACTRN12623000968673.

## 1 Introduction

Many older people live with chronic health conditions, with the prevalence and number of chronic health conditions rising with increasing age (1–3). In addition to chronic health conditions, many older people also report loneliness and social isolation (4). The Global Initiative on Loneliness and Connection defines loneliness as a subjective unpleasant or distressing feeling of a lack of connection to other people, along with a desire for more, or more satisfying, social relationships (5). Loneliness is different to social isolation, defined as having objectively fewer social relationships, social roles, group memberships, and infrequent social interaction (5). Prolonged periods of social isolation and loneliness can negatively impact a person’s mental, physical and social wellbeing, leading to increased risks of developing social anxiety, clinical depression and suicidal ideation (6) and is associated with developing dementia (7, 8), cardiovascular disease (9) and early mortality (10). This may have been compounded by recurrent COVID-19 pandemic lockdowns (8, 11). Susceptibility to depression and anxiety increases with the cumulative impact of social isolation and loneliness on an individual’s mental health and social wellbeing (12, 13). Currently, healthcare systems are focused on treating illness and disease, rather than adopting a preventative approach that enables people to keep healthy and well, such as supporting social connections (6, 14–16). Addressing social needs is critical not just for improving health and wellbeing, but also to ensure improved, appropriate, and efficient use of finite healthcare resources; as older people who are lonely are more likely to seek medical attention in order to satisfy social needs (17). The subsequent long-term impact of unaddressed loneliness on health (7, 9, 15) will further increase healthcare system utilisation (18).

Optimal healthcare delivery is holistic, following the biopsychosocial model, considering social, psychological, and biological factors (19). This approach is designed to enable care that meets the needs of the individual, including enabling continuity of care, with early medical and psychosocial intervention to prevent escalation to more significant health issues (20). Unfortunately, holistic care is lacking, with current models of care being siloed and predominantly focusing on biomedical aspects (14, 16). Loneliness has been found to be more detrimental for mental health correlates and social isolation for physical health (21, 22) but the presence of both loneliness and social isolation further exacerbates poor health outcomes and increases mortality (21, 22).

One intervention that has the potential to address both social isolation and loneliness is social prescribing (23), defined as:

*A means for trusted individuals in clinical and community settings to identify that a person has non-medical, health-related social needs and to subsequently connect them to non-clinical supports and services within the community by co-producing a social prescription – a non-medical prescription, to improve health and wellbeing and to strengthen community connections* (24).

Social prescribing is currently receiving increased interest; however robust evaluations are limited.

To date, existing studies on effectiveness of social prescribing interventions are of varying quality, where a rapid review found mixed results with some positive, mixed and negative outcomes reported (25). Other reviews identified that the majority of studies focus on positive qualitative outcomes (23, 26). There have been limited quantitative outcome studies, focusing mainly on health-related outcomes, showing inconsistent results (23, 26). This may be because the quantitative measures used for evaluation of outcomes may not adequately capture more complex outcomes, such as community connectedness, social engagement, confidence, willingness to give and receive peer-support, and confidence to access services and self-determination and self-care. These ‘hard to quantify concepts’ were captured in qualitative studies, which predominantly reported positive outcomes for participants (23, 26, 27). Additionally, only a few social prescribing interventions measure loneliness (28).

In the UK, the National Academy for Social Prescribing has undertaken systematic reviews on social prescribing and social connection activities with international evidence (23, 29). Existing evidence highlights more robust research is needed and suggests:

a) The most effective models comprise a collaboration of local partner organisations working together;
b) Social prescribing can have a positive immediate impact on a wide range of outcomes, including reductions in loneliness, and improvements in mental health, social connections and overall wellbeing;
c) Social prescribing can reduce pressure on primary care and save healthcare costs;
d) Social prescribing generates a favourable Social Return on Investment (SROI) in most cases;
e) More research is needed that includes more diverse populations; and
f) There is less evidence on the medium and long-term impact of social prescribing, and research in this area is required.

Recent systematic reviews findings have indicated that future research has to include evaluations on intervention outcomes at the individual, community and system levels, implementation outcomes and cost effectiveness (30, 31).

Specific to health economics, there is a need to understand and quantify the social and economic value that community-based assets generate, for example, whether enabling reciprocity and building mutual trust amongst community members promotes social wellbeing that leads to cost saving by reducing escalation of health issues requiring health service use. Loneliness is associated with a substantial economic burden, where individuals who are lonely are more likely to seek medical attention to satisfy social needs (17, 18). A report from 2021 (32) estimated the cost of loneliness at AUD$2.7 billion each year, which equates to an annual cost of AUD$1,565 for each person experiencing loneliness. Therefore, interventions that aim to alleviate loneliness are likely to be cost saving and cost-effective but there is currently limited evidence that this is the case (33, 34).

To enable the delivery of an evidence-based, person-centred approach to social prescribing in Australia, we have codesigned a new program, *Connect Local,* based on current UK models.

### 1.1 The *Connect Local* program

*Connect Local* is a newly-developed, codesigned program where community members living in one south-eastern metropolitan Melbourne local government area (LGA), will be supported to connect with local social services and/or activities through a paid trained Community Connector role, with the aim of reducing loneliness, social isolation and/or depressive symptoms; and improving wellbeing (35). This is a whole of community approach to promote social connection incorporating social prescribing, in addition to network building, awareness raising, volunteer and peer support, and other social capital building activities. The program will be evaluated, considering program effectiveness, cost-effectiveness, and implementation summative and process outcomes.

The *Connect Local* initiative represents a collaboration led by an aged and community care provider, Bolton Clarke; with a tertiary healthcare provider, Alfred Health; a primary care organisation, South Eastern Melbourne Primary Health Network; and a multi-sector and multi-discipline network, the Australian Disease Management Association, called Connecting Communities to Care. Stage 1 comprised co-designing a community-wide approach to facilitate older community members to link with local social supports in one LGA of Eastern metropolitan Melbourne, Australia.

Stage 2 involves implementing and evaluating the *Connect Local* early intervention program that links older Australians with local social supports, in the Glen Eira LGA.

### 1.2 Theoretical framework

In this project, we aim to evaluate a program that will support older community members with at least one chronic condition who are at risk or experiencing loneliness, social isolation and/or depressive symptoms to optimise their wellbeing through building their capacity to socially connect. This will be done by enabling their access to local, relevant, social supports using a paid Community Connector role, supplemented with volunteer and peer support to help them do this.

**Figure 1:**
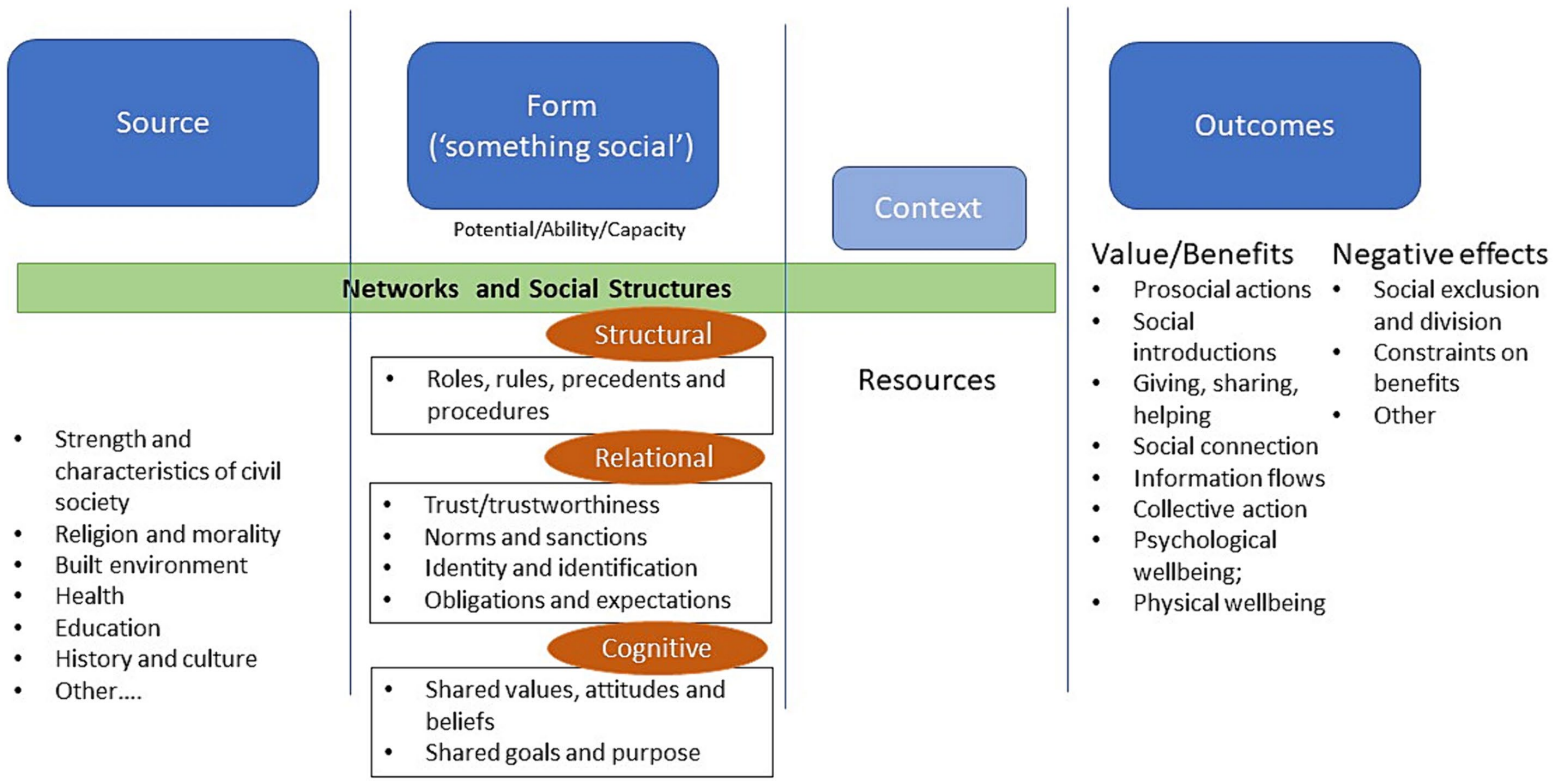
Social capital approach adapted from a model developed by Claridge (43) from the Institute for Social Capital.

Social capital theoretical constructs will underpin this work. Social capital is a broad, umbrella concept with many components and difficult to define, attempting to map the value of relationship networks (36). Basically, social capital is ‘something social’ (called “*Form*”), drawn from a *Source* (be it individual competencies, history and culture, education and others) that has the potential/ability/capacity to produce *Outcomes* that are productive, beneficial and important (36–39). Social capital exists between people, in groups and communities and in communities and society, and can benefit individuals, the collective and/or both. The ‘something social’ requires a structural dimension be in place to enable cognitive and relational dimensions to occur (36). More specifically, the structural category *facilitates* cooperation (enabling it to occur), but the cognitive category *predisposes* cooperation (so people actively seek out and participate in social engagement) (40). These three dimensions are connected and mutually reinforcing, so cannot be treated separately. In addition, the context within which these activities are implemented is important, which includes the resources necessary to enable them to occur. Resources can be defined as information, trust, support (41), as well as the traditional wealth, status, power and social ties linked to the individual (42) – context (including resources) mediate the ability to achieve the desired outcomes. Our approach focuses on the pro-social aspects (giving, sharing, helping, caring, supporting) provided within a network of social connection, which will lead to reduced loneliness, social isolation and depressive symptoms and improved psychological and physical wellbeing. All of which will eventually lead to reduced health system use (such as reduced hospitalisations). Putting this all together, the social capital approach that we will utilise can be depicted in Figure 1.

Specific to loneliness, we refer to the conceptual model developed by Lim et al. (44) which guided the different types of variables collected in our evaluation but also provide a more comprehensive socioecological approach to reducing loneliness. The Conceptual Model of Loneliness articulates the different risk factors and correlated for loneliness in three parts: demography (i.e., age, gender, marital status, living states, socioeconomic status), health (physical, mental, cognitive, brain and biology), and socio-environmental (e.g., workplaces, digital use) (44). It is assumed that everyone holds at least some risk factors of loneliness and these risk factors interact and may lead to problematic levels of loneliness (44). Therefore, it is critical to consider how different types of factors (such as individual, relationship, and community factors) can also contribute to the severity of loneliness.

**Figure 2:**
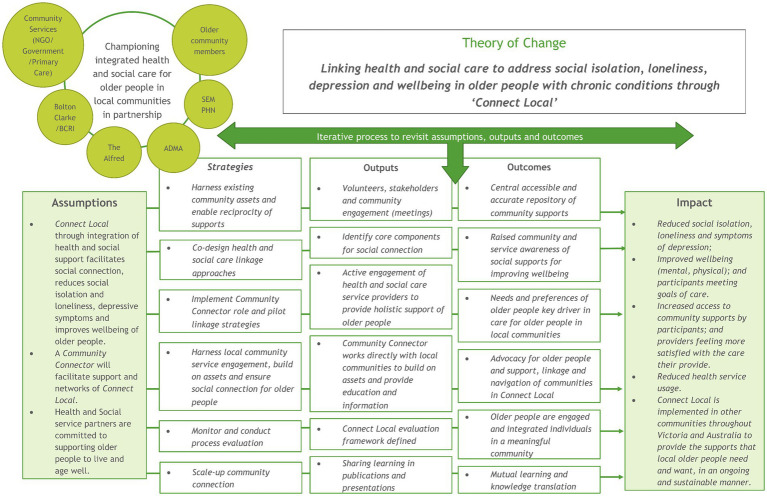
Theory of change for “*Connect Local*”.

Finally, program development was underpinned by a Theory of Change, generated according to the changes stakeholders aspired to achieve as a consequence of delivery of programs to promote holistic wellbeing, shown in Figure 2. Table 1 shows the participants of the stakeholder engagement and level of engagement that led to the development of the Theory of Change. Participants included those involved in codesign from previous studies aiming to promote holistic wellbeing through community supports: Older Women Living Alone (OWLA) (45) and Peer support for Older WomEn to pRomote wellbeing and independence (POWER) (46). These elements were then refined from codesign sessions in Stage 1 of this study.

**Table 1:** Participants involved in codesign that refined the theory of change and their engagement.

Participant group	Descriptors of participants	Engagement
OWLA		
Older women living alone	*N* = 13. 100% women, average age 72 (±8.7) years, 10 (77%) Australian born.	Three in person sessions.
Advocacy and social service representatives	*N* = 11. 10 (91%) women. Representatives from: aged care and community provider (*N* = 6), social care provider (*n* = 1), tertiary care provider (*n* = 1), city council representative (*n* = 1), primary healthcare network representative (*n* = 1), community health service provider (*n* = 1)	Three in person sessions.
POWER		
Older women living alone	*N* = 5. 100% women. Average age 76.2 years, 2 (40%) Australian born.	Four in person sessions – one combined with volunteers.
Peer support volunteers	*N* = 7. 100% women, average age 72.1(±8) years, 100% born in Australia.	Three in person sessions – one combined with older women living alone.
Health and social service representatives	*N* = 11. Members from: local government area (*n* = 5), Aged care and disability service (*n* = 1), local health service (*n* = 1), local tertiary hospital (*n* = 1), university representative (*n* = 1), community health service (*n* = 1), age and community care provider (*n* = 1).	One in person session.
*Connect Local*		
Older community members	*N* = 6. 4 (67%) women. Average age 69 years, 3 Australian born, 2 with disabilities and all with at least one chronic health condition.	1 online, 2 hybrid 3 in person sessions.
Social activity/service representatives	*N* = 8. 6 (75%) = women. Members from: local government area (*n* = 1), elder education organisation (*n* = 1), professional retiree group (*n* = 3), community support (*n* = 1), neighbourhood house (*n* = 1), meals on wheels (*n* = 1), police community register (*n* = 1).	3 in person sessions
Health service representatives	*N* = 11. 9 (82%) women. Members from General Practice (*n* = 2), Tertiary Health (*n* = 2), Community Health (*n* = 2), Pharmacy (*n* = 1), State Ambulance Service (*n* = 2) and community ambulance service (*n* = 2)	1 online and 2 in person sessions

### 1.3 Evaluation of *Connect Local*

We propose to contribute to the evidence-base by undertaking an evaluation to measure program effectiveness, health economic outcomes and the implementation of the *Connect Local* program. This evaluation will include medium- and longer-term loneliness, social isolation, depressive symptoms, and wellbeing outcomes of the community-wide social connection initiative with a comparator group. The evaluation will also include a comprehensive economic evaluation, using a Social Return on Investment (SROI) approach (47), to cover outcomes at the individual, community and system levels. Finally, implementation outcomes will also be evaluated to consider Reach, Adoption, Feasibility, Acceptability, Appropriateness, Fidelity and Sustainability of the intervention.

At the end of the project we aim to contribute to the greater understanding of how a social connection program for older people functions, building on the middle conceptual and grand theories used to underpin this work. We anticipate this will enable a greater conceptualisation of how implementation of this program impacts the included stakeholders and is in turn impacted by the multiple contextual components.

## 2 Methodology

The research team is using a pragmatic approach, where all necessary approaches will be used to understand the research problems. There are a number of different components that will be considered, and each component will be using a different approach.

### 2.1 Individual and cost effectiveness outcomes

The outcomes related to the impact of the *Connect Local* intervention on older individuals and cost effectiveness will include a post-positivist ontological approach, where there is a single reality, imperfectly known (48, 49). Following on from this, our epistemological approach considers that we can only establish probable truths, as obtaining knowledge is subject to human error (48, 49). Axiomatically, we are considering that our intervention will make the community a better place by reducing loneliness, social isolation and/or depressive symptoms, leading to wellbeing, increased community activity/program use and reductions on health system use. The evaluation team aims to observe and measure the changes resulting from the *Connect Local* intervention, therefore position themselves outside the context of the intervention.

### 2.2 Community, health system and implementation outcomes

The outcomes related to the impact of the *Connect Local* intervention on the community, health system and the implementation of the program will be using a critical theory approach, where there are multiple subjective realities, influenced by power relations in society (48, 49). The knowledge is subjective, and co-constructed between individuals and groups (48, 49). The aim is to understand the relationships and these groups. The evaluation team is part of the implementation process, actively engaging and therefore position themselves inside the context of the intervention.

### 2.3 Researchers background

The ten members of the research team (nine female), consist of eight researchers, with research experience varying from early career (E.R., K.F., and S.A.) midcareer (R.O., D.F.), to highly established (M.L., L.E, and J.L.). The two non-research team members have work roles that support delivery of care in health (A.Y) and aged and community (K.R.) systems.

Five team members currently work within an aged and community care service organisation (R.O., K.R., E.R., S.A., and J.L.). Two team members work as clinicians, one in a hospital (D.F) and one in primary care with a university position (M.L.). One works within a university environment only (L.E.) and one team member works at a peak health organisation (K.F.). The professional backgrounds of the research team are also diverse, with clinical training of six team members [in the fields of podiatry (R.O.), optometry (S.A.), nursing (K.F.), psychology (M.L.), speech pathology (J.L.), endocrinology and general medicine physician (D.F.)] and fields of health economics (L.E.) and business and management (K.R. and A.Y.).

## 3 Methods

This manuscript includes the relevant elements from the Standard Protocol Items for Clinical Trials (SPIRIT) guidelines (50), and the consolidated criteria for reporting qualitative research COREQ (51) and Standards for reporting implementation studies (STARI) (52).

The evaluation will be a Type 1 Hybrid effectiveness-implementation study design (53), with research objectives to ascertain program effectiveness, program cost-effectiveness; and implementation summative and process outcomes. This evaluation comprises Stage 2 of a two-stage project, with Stage 1, codesign of the program, being published separately. Hypotheses and research questions in the form of PICO [participants, intervention, comparator and outcome(s)] (54) have been generated for the research components involving a comparator group for quantitative data. Propositions and research questions in the form of SPIDER (Sample, Phenomenon of Interest, Design, Evaluation, Research type) (54) have been generated when data is mixed method or qualitative.

Hypotheses are proposed for the first two evaluation components:

### 3.1 Program effectiveness

Compared to the comparator group, older people receiving the intervention will, when compared to baseline, report at 3-, 6- and 12-months follow-up:

(i) a reduction in loneliness, social isolation and/or depressive symptoms;
(ii) improvement in wellbeing and quality of life;
(iii) increased access to activities/services in the community.

Three months after participation in the program, community volunteer participants will have improved wellbeing and quality of life when compared to baseline.

It is hypothesised that all stakeholders: intervention, volunteer and service provider participants will also have a positive perception of and experience with the program.

*Research question for older participant cohort using PICO:*

Does participation in the *Connect Local* social connection program reduce loneliness, social isolation and/or depressive symptoms, improve wellbeing and quality of life and increase access to activities/services in community of older Australians when compared to a similar group who do not have access to a similar program?

*Research question components for stakeholders using SPIDER:*

Sample: volunteers and service provider participants;

Phenomenon of interest: perception and experience of program;

Design: prospective surveys and semi-structured interviews;

Evaluation: impact on community and health services, and perceptions of the program;

Research type: mixed methods.

Question: What are the perceptions and experiences of volunteers/service provider participants after being a part of the *Connect Local* program?

### 3.2 Program cost-effectiveness

(i) Among the target cohort within the study LGA, *Connect Local* will generate improvements in the health, wellbeing, and quality of life of participants that can be translated into monetary values. This will be established by comparing the situation before and after the *Connect Local* intervention and comparing outcomes with the comparator group.
(ii) When comparing the cost of the intervention with the social and economic outcomes, including health service utilisation, the SROI ratio will yield a return greater than the investment.

*Research question using PICO:*

Is *Connect Local* more cost effective to address loneliness, social isolation, depressive symptoms and wellbeing in older community members compared to no *Connect Local*?

A proposition is proposed for the third research component:

### 3.3 Implementation: *Connect local* will be implemented as planned

The program effectiveness and cost-effectiveness components will be evaluated in a prospective, cohort quasi-experimental (non-randomised), pragmatic trial, using a convenience sample of participants, with the intervention group drawn from those living in the target LGA who access the community connector.

*Research question components using SPIDER:*

Sample: older community members, volunteers and service provider participants;

Phenomena of interest: reach, adoption, feasibility, appropriateness, fidelity, sustainability.

Design: prospective surveys, semi-structured interviews, administrative data;

Evaluation: implementation of program as planned;

Research type: mixed methods.

Question: What are the perceptions and experiences of [participants] about the *Connect Local* program and it being implemented as planned?

### 3.4 The intervention: *Connect Local*

The *Connect Local* program will involve eligible individuals to access a trained and paid Community Connecter professional. Community Connectors are individuals with a counselling, community development, health/allied health professional and/or life coaching background, who receive training in social prescribing (55), wellness and reablement and positive ageing, diversity in ageing, mental health first aid and trauma informed care.

**Figure 3:**
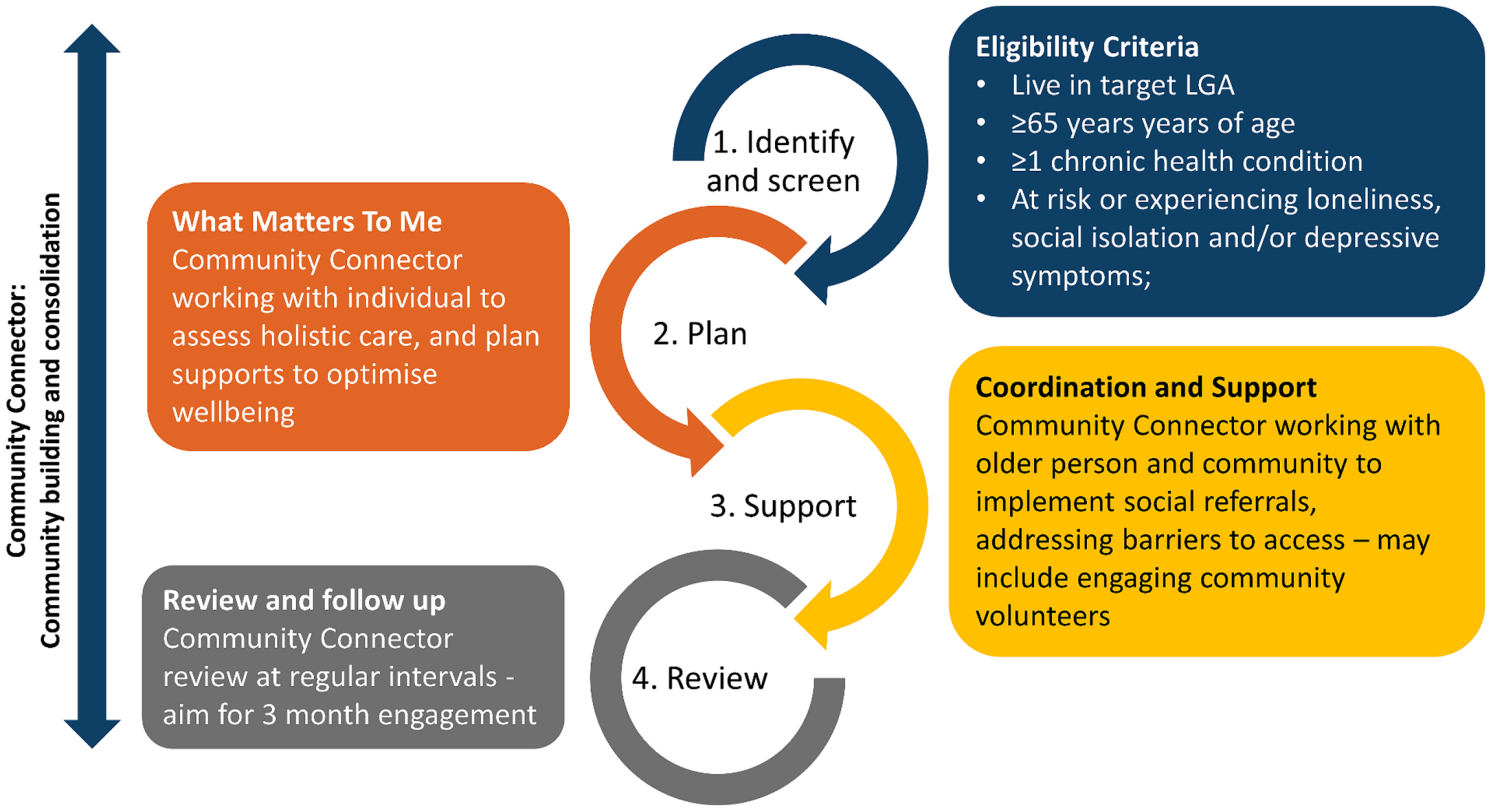
Service flow of *Connect Local*. Adapted from Mann et al. (20).

Once a referral/contact is received, the *Connect Local* program manager contacts the community member and first discusses the program, to ascertain that it is what they are seeking. If so, the community member is screened for eligibility, namely, they live in target geographic location, are aged 65 or older, have a chronic health condition, and are in need of social connection. If appropriate, the program manager organises the Community Connector to contact the individual to organise a time to meet face to face and commence the program. As shown in Figure 3, the Community Connector will work with eligible community members to:

1. **Identify** what matters to the individual and screen for loneliness, social isolation and depressive symptoms: the Community Connector holds a rapport-building conversation, involving open-ended questions and using motivational interviewing techniques guided by the NHS document ‘What matters to me?’ (56). The question topics have previously been codesigned with Community Connectors and community members to ensure they are fit-for-purpose for the local population, and include: expectations of the program, personal history in the area, past activities of interest, and when they remember being happy – where they were, who they were with, and what they were doing;
2. **Work together** on generating goals that address a social need/s, and a develop a **plan** to achieve these goals by linking the individual to activities and/or services being offered in the target geographic area. This is based on the discussion on “What matters to me?” and what existing available programs and activities are in the region, as well as the community member’s level of digital engagement and any transportation requirements. Usually three or four programs/activities are generated for pursuing;
3. **Support** the individual to access these activities/services using local resources such as volunteer support including transport. This involves the Community Connector reaching out to program/activity providers agreed to in point 2, and organising to meet with the community member at the program/activity location. This serves as an introduction and, if possible, have the community member participate or at least view the program/activity, to ascertain whether this is something they are interested in engaging with. If the community member is interested, the Community Connector facilitates ongoing engagement in the program/activity, as needed. If the community member is not interested in this program/activity, they pursue an alternative program/activity discussed previously; and.
4. **Review** follow up to ensure that the activities and/or services are addressing their needs. Should there be any issues, the Community Connector may either engage with the service/activity provider to address them or work with the individual to develop a new goal, plan and access new activity/service.

Individuals who participate in all four elements will be considered to have completed the full program. Meetings between the Community Connector and the participant will be undertaken in a mutually agreed upon location, including local community houses, local spaces such as libraries, local cafés, or in the participant’s home.

People will be engaged in the program through multiple sources: the local tertiary hospital, aged care provider, general practices, allied health providers, community health providers, through the program website, and general community access.

#### 3.4.1 Comparator group (usual care)

These individuals will receive usual care (regular access to activities in their LGA), and will not be engaged in the *Connect Local* program.

### 3.5 Participants of the evaluation

There are three participant groups involved in this evaluation: older community members, community volunteers, and health and social care providers.

#### 3.5.1 Older community members: intervention group

The inclusion criteria include being aged 65 years or older; understand and speak English sufficiently to understand and be involved in the program; live in the target LGA; community dwelling (including those experiencing homelessness); have at least one chronic health condition; are at risk or experiencing loneliness, social isolation and/or depressive symptoms. Screening tools are summarised in Table 2, loneliness will be assessed using both the Single Item Measure and UCLA-3 as per recommended national indicators of loneliness (66).

**Table 2:** Intervention outcome evaluation methods.

Evaluation component *Definition*	Indicators	Methods and tools
Individual level *Impacting individuals directly*	*Older person participants:*	
	Loneliness	Single-item measure of loneliness: How often do you feel lonely?, responses: often or always, some of the time, occasionally, hardly ever or never (often or always, some of the time indicating loneliness)	UCLA-4 (57)
UCLA-3 (score of ≥6 indicates at risk of loneliness) (58)
Social isolation	Lubben-6 (score ≤ 12 indicates at risk for social isolation) and − 18 (59);
Social anxiety	Mini-SPIN (Social Phobia Inventory, SPIN) (60);
Depressive symptoms	GDS-5 (score of ≥2 is indicative of depression) (61);
Wellbeing and quality of life	Physical wellbeing (and QoL): EQ-5D-5L (62); Mental wellbeing: Warwick-Edinburgh Mental Wellbeing Scale (WEMWBS) (63);
Goals attained^+^	Goal Attainment (64)^+^.
Perceptions of program^+^	Interviews on perceptions of the program^+^.
*Service providers:*	
Perceptions of program	Interviews/focus groups on perceptions of the program.
*Volunteers:*	
Wellbeing and quality of life	Physical wellbeing (and QoL): EQ-5D-5L (62); Mental wellbeing: Warwick-Edinburgh Mental Wellbeing Scale (WEMWBS) (63);
Perceptions of program	Interviews/focus groups onperceptions of the program.
Community level *Impacting broader community*	*Older person participants:*	
	Access to activities/services Number and types of services offered to participants	Resource use questionnaire [adapted from Fletcher et al. (65)]
	*Community service providers:*	
	Impact of program on community services	Community service impact survey [adapted from NHS (56)]
	Community service provider Satisfaction with program	Interview/focus group with community service providers
Health system level *Impacting at the health service level, including hospitalisations, GP visits and other health service visits.*	*Older person participants:*	
	ED presentations; Hospitalisations; Hospital length of stay Number of GP consultations Number of GP care plan reviews	Resource use questionnaire [adapted from Fletcher et al. (65)]
	*Health Service providers:*	
	Perspectives of staff in general practice and other referral agencies Health provider satisfaction	Interviews/focus groups

^+^Data not being collected from community members in comparator group.

The exclusion criteria include living in residential aged care; individuals who do not speak English sufficiently to understand the program; and individuals who do not have the cognitive capacity to consent [assessed using a Cognitive Capacity to Consent Checklist (67)].

All eligible individuals will be made aware of the evaluation by the Community Connector and asked if their details can be shared with the researchers to contact them about the study. The researchers will contact the individuals to obtain consent. If consent is obtained, individuals will be asked if they would like to be informed of trial results.

#### 3.5.2 Older community members: comparator

Given the proposed whole of community impact of *Connect Local* program, the inclusion criteria for the comparator group are the same as for the intervention group, except that they do not live in the target LGA. These participants will be recruited from the local tertiary hospital and aged and community care provider. All eligible individuals will be made aware of the program through their care provider and will be asked if their details can be shared with the researchers to contact them about the study. The researchers will contact the individuals to obtain consent. Recruitment will be monitored in blocks of five to match comparator participants by age and gender to the intervention participant group.

#### 3.5.3 Community volunteers

Inclusion criteria include: any age (including younger people of school age); Live, work or are willing to regularly come to the target LGA; currently a volunteer engaging with community members receiving *Connect Local* program; and understand and speak English sufficiently to understand the program.

Organisations engaging volunteers will share the information on the evaluation, and all volunteers who have been involved in supporting individuals engaging in *Connect Local* will be invited to reach out to researchers and participate in the evaluation.

#### 3.5.4 Health and social service providers

Inclusion criteria are individuals who work at health and social services that support eligible older participants or volunteers in the target LGA, including the *Connect Local* program and the Community Connectors. Researchers will obtain information on health and service providers engaged in the program from the Community Connector and contact them directly to ask them to participate in the evaluation.

### 3.6 Data collection

#### 3.6.1 Procedures

Data will be collected either by researchers via the phone, in-person, in paper-based surveys, or by Community Connectors during program operations. Study data will be collected and managed using REDCap electronic data capture tools hosted on a secure server at Bolton Clarke (68), which only researchers will have access by password. Table 2 outlines the different levels and the data collected, while Table 3 outlines the time points of data collection. If data collection causes distress to participants, interviewing will be immediately halted and existing escalation procedures will be followed, including referral back to the referring healthcare provider (community members), service responsible (volunteers), or external mental health services.

**Table 3:** Schedule of enrolment, interventions and assessments, per SPIRIT guidelines (50).

	Study period				
Timepoint:	Screening	Baseline	3-months	6-months	12-months
Enrolment					
Screening	X				
Informed consent	X				
Intervention					
*Connect Local* intervention		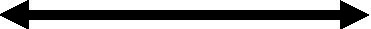			
Usual care (no intervention)	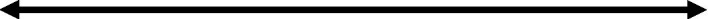				
Assessments					
Single-item measure of loneliness (69)	X				
UCLA – LS 3 (58).	X	X	X	X	X
Lubben Social Network Scale (LSNS-6) (70).	X				
5 item Geriatric Depression Scale (GDS 5) (61).	X	X	X	X	X
Demographic information		X			
UCLA-LS4 (71).		X	X	X	X
Lubben Social Network Scale (LSNS-18) (59)		X	X	X	X
Mini-Social Phobia Inventory (Mini-SPIN) (60).		X	X	X	X
Warwick-Edinburgh Mental Wellbeing Scale (WEMWBS) (63);		X	X	X	X
EQ-5D-5L (62);		X	X	X	X
Goal attainment (64).		X	X	X	X
Community service impact survey				X	X
Resource use questionnaire		X	X	X	X
Perceptions (acceptability), use and satisfaction with program			X		
Process data			X	X	X
Perspectives of staff in General practice and other referral agencies			X	X	X

#### 3.6.2 Outcomes: intervention

The aim of this evaluation is to assess the participant outcomes, and program effectiveness and impact from the Individual, Community and Health Service perspectives.

##### 3.6.2.1 Individual level

This is the level at which individuals are impacted directly, including older community members, volunteers and health and social service providers.

For older community members, we will consider the primary outcome as a mean change in loneliness, from baseline at 3, 6 and 12 months for participants receiving the *Connect Local* program, when compared to the comparator group. This will be measured using UCLA-3 as informed by previous research that support the sample size calculations (58). At this stage, there is insufficient research data to calculate sample size when using UCLA-4 (57), however this tool has been recommended for use by community organisations by Australia’s national network, Ending Loneliness Together (71), therefore we will also utilise this tool to capture loneliness to ascertain whether it can be utilised with this cohort in future studies.

As outlined in Tables 2, 3, secondary outcomes, including social isolation, social anxiety, depressive symptoms, and improvement in quality of life and wellbeing, are a mean change from baseline at 3, 6 and 12 months for participants receiving the *Connect Local* program, when compared to the comparator. Achieved goals, as measured by the Goal Attainment scale (64), will also be gathered from the intervention group.

For community volunteers involved in the *Connect Local* program, as per Tables 2, 3, we will collect health-related quality of life and wellbeing measures at baseline, 3, 6 and 12 months.

Perceptions of the program will be collected from community members in the intervention group, volunteers and service providers involved in referred to the *Connect Local* program through interviews or focus groups at three months from participant baseline. Questions will invite participants to share their thoughts around the following aspects of the program: Relational (trust and trustworthiness, obligations and expectations, identity and identification), Cognitive (shared language, codes and narratives; shared values, attitudes and beliefs; and shared goals and purpose), and Structural (roles, rules, precedents and procedures).

##### 3.6.2.2 Community level

As per Table 2 the following indicators will be collected to evaluate the impact of the program on the broader community: the number of participants who access community activities/services, the number and types of services offered to and used by participants in both the intervention and comparator groups at baseline, 3, 6 and 12 months using the Resource Use Questionnaire [adapted from Fletcher (65)].

We will also ask community service and/or activity providers about the impact of the program on their services six monthly, using the Community Service Impact Survey (72), and their perspectives of the program through interviews or focus groups at three months from when participants were engaged in their program.

##### 3.6.2.3 System level

To evaluate impact of *Connect Local* on health services use such as hospitalisations, General Practitioner (GP) visits and other health service visits, several health service-related indicators will be collected from participants in both the intervention and comparator groups at baseline, 3, 6 and 12 months using the Resource Use Questionnaire [adapted from Fletcher (65)], as per Tables 2, 3. Using interviews, the service provider morale in general practice and other referral agencies and healthcare provider satisfaction will also be gathered at three months from when their patients engaged in the *Connect Local* program.

###### 3.6.2.3.1 Sample size calculation

The primary outcome is a reduction in loneliness at 3 months for participants receiving the *Connect Local* program, when compared to the comparator group. A program conducting similar activities found a statistically significant reduction in mean UCLA-3 scores of 0.85, with a medium effect size of 0.37 (73). Using this effect size at 0.05 alpha, 76 participants are required in the intervention group to reach 0.9 power. Allowing for 15% attrition at the 3 month follow up, we have increased the sample size to 88. A matched comparator group will be recruited at a 1:1 ratio, as such a total of 176 participants will be recruited to the intervention and comparator groups.

#### 3.6.3 Outcomes: cost-effectiveness

To consider the social value generated by the initiative, a triple bottom line of social, economic and environmental value through Social Returns on Investment (SROI) will be considered (74). Economic evaluation is a tool used to guide resource allocation decisions in health care, where effects are often expressed in health-related units. However, it is anticipated that interventions that alleviate loneliness are not only associated with health benefits but also broader societal benefits, where benefits often accrue across sectors. As such, a broader evaluation framework is required to determine the social and economic value of the *Connect Local* intervention, such as Social Return on Investment (47).

The scope of the health economic evaluation will involve data collected as part of this evaluation between May 2023 and May 2025 (8 quarters), during which time the program will be fully established and running in the target LGA. The categories of stakeholders that will be operationalised will be:

1. Beneficiaries: those who experience the outcomes of an intervention (community members) involved in *Connect Local*;
2. Implementers: suppliers and subcontractors (Bolton Clarke, Social Service providers);
3. Promoters: those who provide support and a conducive environment for implementation of the intervention (health care providers); and
4. Funders: those who directly and indirectly finance the project (The Ian Potter Foundation, Department of Health, target LGA City Council).

The theory of change, shown in Figure 3, developed for this study, was used to underpin this cost-effectiveness evaluation.

The evaluation component will involve the following SROI process steps:

##### 3.6.3.1 Evidencing outcomes and giving them a value

Data on loneliness, social connection, depressive symptoms, wellbeing, quality of life and health service use at baseline, 3, 6 and 12 months will provide evidence of outcomes, drawing comparisons between the intervention and comparator groups. Each outcome will be then monetised using financial proxies. Costs and benefits that occur at different time points will be made comparable by adjusting for inflation in order to calculate net present value (47).

##### 3.6.3.2 Establishing impact

This stage will determine those aspects of change that would have happened anyway or are a result of other factors. Such aspects will be eliminated from consideration. Qualitative and quantitative data (surveys on loneliness, social connection, depressive symptoms, wellbeing and quality of life at baseline, 3, 6 and 12 months) will provide this evidence. Monetised outcomes will be discounted on the basis of what would have happened without the intervention (deadweight), what outcomes are displaced by the intervention (displacement), who else has contributed to the outcomes aside from the funder (attribution), and whether experience of the outcomes declines over time (drop off).

##### 3.6.3.3 Calculating the SROI

This will involve adding up all benefits. Investment into the *Connect Local* will be compared to the discounted, monetised value of benefits. The discounted, monetised value of benefits and outcomes will be divided by total investment (inputs) to estimate the SROI ratio.

#### 3.6.4 Outcomes: implementation

In order to ascertain the effectiveness of the implementation of the program, the following methods will be used:

##### 3.6.4.1 Reach and adoption

To ascertain the useability of the intervention (does it reach the right people? Are they using it?), we will collect demographic data of participants, number of clients who engage with the Community Connector and number of participants who complete the program, using administrative data from the *Connect Local* program, stored in Bolton Clarke’s client record system.

##### 3.6.4.2 Feasibility, acceptability, and appropriateness

To ascertain the perceived fit and relevance of the intervention, the extent to which it can be used in the participating organisations, and acceptability (overall experience), we will collect: time taken to complete the program (collected through administrative data), perception/attitudes of all stakeholders as to the ease of use, usefulness of, and satisfaction with, the program (through interviews), and organisational support systems and processes also through interviews as well as the Resource Use Questionnaire [adapted from Fletcher et al. (65)] collected at 3, 6 and 12 months.

##### 3.6.4.3 Fidelity and sustainability

To ascertain the uptake of the intervention into practice as planned, and the extent to which it can be embedded into practice to promote sustainability, we will use administrative data on the delivery of all four program components outlined in section 2.2 above, and organisational support systems and processes to enable this. We will supplement this information with in-depth data from interviews/focus groups with all participant groups, using open-ended questions around the participant experiences of the program components by participants. These questions will include sharing thoughts on: being made aware of the program and signing up; the engagement with the community connector – the process of finding out ‘what matters to you?’; How they connected with the programs/activities; Whether this went well and what happened then.

### 3.7 Data analysis

#### 3.7.1 Outcome 1: intervention evaluation – program effectiveness

##### 3.7.1.1 Quantitative data analysis

For all quantitative, repeated, continuous individual level variables (loneliness, social isolation, social anxiety, depressive symptoms, wellbeing, quality of life) the main research question will be whether the study intervention is effective at improving the outcome of interest compared with usual care. Modelling these data will focus on fitting linear mixed models using different covariance matrices such as: unstructured, an exchangeable-changeable and AR level-1 residual matrices, where the mean structure of all fitted models include a term for intervention group and a term for time and an interaction term for time and intervention. Non-linear outcomes will be fitted using Generalised Estimating Equations (GEE) analysis which requires that the missingness mechanism is Missing Completely At Random (MCAR). Alternatively, polynomials and/or piecewise linear functions will be utilised for modelling outcomes with non-linear growth trajectories if responses are Missing At Random (MAR). To ascertain whether the missing data is MCAR or MAR, we will utilise methods such as data inspection, applying domain knowledge and comparing summary statistics between cases with complete data and those with missing data to determine the missing data mechanism. An understanding of the mechanism will enable an appropriate statistical method to be chosen to handle the missing values in the data set.

For quantitative, binary or dichotomous individual level outcomes (scales that have cut-off scores: loneliness (UCLA-3), social anxiety, depressive symptoms) additional analysis will be undertaken addressing the research question: do the interventions differ in their effectiveness, such that individuals in the intervention group experience a greater improvement in their probability of achieving the desired outcome compared to those receiving usual care. Generalised linear mixed models (GLMMs) for dichotomous outcomes will be used as well as GEE. The typology of missing data will be investigated to make sure that the assumptions of the adopted statistical technique are not violated. Our choice of GLMMs is based on the specific characteristics of the data, as well as the advantages provided by GLMMs in handling dependent responses in longitudinal or repeated measures studies. As highlighted in Breslow and Clayton (75), GLMMs incorporate random effects into the linear predictors and are particularly well-suited for modelling the dependence among response variables in such study designs.

Selection of the model that best fits the data will be based on the low goodness-of-fit measures such as the Akaike information criterion (AIC) and Bayesian information criterion (BIC). Additionally, the log-likelihood ratio test will be employed as another method for evaluating model fit.

All quantitative community and system level variables, as well as the goal attainment will be analysed descriptively. Descriptive statistics will be presented as proportions, means (standard deviations) or, for variables that did not conform to a normal or log-normal distribution, medians (interquartile range).

Statistical analysis will be performed using STATA V.15.0 (STATA Corp LP., College Station, Texas, United States). We will use a Type I error rate of 5% to indicate statistical significance, with 95% confidence intervals (CI).

###### 3.7.1.1.1 Missing data

General and generalised linear mixed models use maximum likelihood estimation which produces robust/unbiased estimates as they make implicit corrections for missing data, hence they are likely to retain more power if participants are lost to follow-up than traditional repeated-measures ANOVA/ANCOVA approaches which use “complete-case-analysis” approach that only includes cases with no missing data in the analysis. Maximum likelihood estimation (MLE) for incomplete longitudinal data (continuous or categorical) uses all available information, which means all participants will be included in the analysis regardless of whether they had complete responses for all occasions/timepoints or not. The MLE estimates are consistent if the assumption of data missingness is MAR and is more robust than using imputed values.

If more than 5% of the outcome data are missing, a sensitivity analysis will be conducted comparing fitted models in terms of estimates and corresponding standard errors using maximum likelihood estimation with and without considering different imputation techniques.

##### 3.7.1.2 Qualitative data analysis

For all qualitative individual, community and system level variables, interviews with participants will be audio-recorded, transcribed and then analysed utilising thematic analysis. Thematic analysis based on grounded theory involves finding repeated patterns of meaning within qualitative data (76) and will be facilitated with the use of qualitative management and analysis software such as NVivo (77). In addition, an interview summary will be created for each interview and circulated to the research team, facilitating the team’s ongoing knowledge of the data being collected. An inductive approach will be used within the project, which allows for themes and findings to emerge from the data, grounding the findings in the perspectives and experiences of participants. Finally, situational analyses will be used to supplement basic grounded theory with situation-centred approaches, enabling the consideration of all of the collective actors and the arena’s within which they engage to develop and fully articulate an ordered situational map (78).

###### 3.7.1.2.1 Mixed method approach

Triangulation will be used to combine the qualitative and quantitative data using a deductive-simultaneous design (79) where the core component is quantitative, and the supplemental component is qualitative.

#### 3.7.2 Outcome 2: health economic evaluation – program cost-effectiveness

The following accepted systematic approach to analyses of the SROI approach will be undertaken (47, 80).

The research team will identify and categorise the stakeholders, and the outcomes most relevant for each, and then apply the following tools of SROI (outlined below): consideration of deadweight, displacement, attribution and drop off. We will do this through discussion between team members undertaking evaluation and program implementation in relation to each of the outcomes considered in the SROI. See the Box 1 re: tools of SROI.

**BOX 1 Tools used to calculate Social Return on Investment (SROI)**

**Tools of SROI**

Establishing impact (attribution, deadweight, displacement, drop-off):

To minimise the risk of overclaiming the benefits it was necessary to account for deadweight, displacement, attribution, and attrition.

*Deadweight:* responds to the question: ‘How much of the outcome would still be attained without the activity delivered?’

We will use the quantitative data captured in the comparator group to ascertain this information, measured as a percentage, and then that percentage of the outcome will be deducted from the total quantity of the outcome.

*Displacement* responds to the question: ‘Were there any activities with the same outcome displaced by the intervention being evaluated?’

We will capture this information when collecting service information from participants involved in the program and comparing them to what was captured in the comparator group – using a percentage and deducted from the total outcomes.

*Attribution* responds to the question; ‘Who else contributed to the attainment of the outcome?

The research team will identify all relevant stakeholders and to assess how much of each outcome could be attributed to the delivery of Connecting Communities to Care with due circumspection. This will be calculated as a percentage.

*Drop-off* responds to the question: ‘How much of the outcome is lost in the years post intervention?’

To calculate the percentage of the outcome lost in the years after delivery of the intervention. It is calculated by deducting a fixed percentage from the remaining level of outcome at the end of each year. This will be calculated as a percentage.

*Calculating the SROI:*

The calculation of the SROI will consider the total value of the inputs in the program. The discounted, monetised value of benefits and outcomes will be divided by total investment (inputs) to estimate the SROI ratio.

#### 3.7.3 Outcome 3: implementation – process evaluation

The process evaluation aims to assess the effectiveness of the implementation process on reach, adoption, feasibility, acceptability, appropriateness, fidelity, and sustainability.

1. Reach and adoption: this will include quantitative data that will be reported descriptively, using frequencies and proportions.
2. Feasibility, acceptability and appropriateness: this will include quantitative data that will be reported descriptively, using frequencies and proportions. Qualitative data will be analysed thematically using an inductive approach, as described above, to ascertain themes around feasibility, acceptably and appropriateness of the program. Process data regarding organisational support systems and processes will also be reviewed by the research team, discussed as a group, considering the content in light of the qualitative data analysis results to form synthesised outcomes. This information will be reported narratively.
3. Fidelity and sustainability: interview qualitative data will be analysed deductively, to ascertain whether program components were delivered as planned. Further, information on organisational support systems and process will be reviewed by the research team to consider the sustainability of the program. This information will be reported narratively.

##### 3.7.3.1 Mixed methods approach

Expansion method, using simultaneous design where the core component is quantitative and the supplemental component is qualitative (79).

## 4 Discussion

The *Connect Local* early intervention model is designed with end users to positively impact the wellbeing and quality of life for an older population at risk or experiencing loneliness, social isolation and/or depressive symptoms, by utilising a social prescribing approach. Our intervention evaluation will determine impacts on community members involved in the program, as well as volunteers and service providers involved, over 12-months. The SROI evaluation will highlight social, economic and health outcomes for the beneficiaries, the healthcare system and wider society. The implementation evaluation will observe and gather information on the implementation of the program to support future implementation of similar programs. Collectively this mixed method evaluation of effectiveness, cost-effectiveness and implementation will add to the evidence base, providing much needed long-term data in a large sample group (30, 31).

The number of people aged 65 years and older is expected to rise (81), and therefore the number of people with chronic health conditions is also expected to rise. The consequences will increase pressure on the health system, with public expenditure on health and social care likely to struggle to meet demands. Therefore, to ensure people with health and social care needs can continue to access appropriate and timely care, we need to address any issues early, before they escalate, and support people to stay well. The World Health Organization is focusing on promoting health and wellbeing (82) including promoting social connection, where human beings adopt a collaborative and equitable approach in their relationships with fellow human beings, building bridges between medicine, public health and social sciences, where programs are codesigned with those who will benefit (82). Social prescribing involves the delivery of more holistic, person -centred care, aiming to empower individuals to take care of their own health and well-being and ultimately reduce stress on health systems. This work will contribute to building the evidence-base through implementation and evaluation of *Connect Local*, an approach using social prescribing.

We anticipate that our *Connect Local* early intervention program will lead to sustainable and life-changing improvements among the participants, particularly on reducing loneliness. By preventing escalation to more significant health issues and using a preventative approach, the model may also reduce the development of associated downstream societal and economic costs. The SROI evaluation and consolidation of our *Connect Local* model will enable decision makers to allocate resources and replicate such evidence-based programs.

We aim to disseminate these findings through multiple avenues including reports to funders, peer reviewed articles and presentations of research findings, and broader dissemination to stakeholders including local older community members, as well as volunteers, service providers and decision makers. We aim to provide robust information to enable decision makers to select and implement this approach more broadly.

## 5 Conclusion

Social prescribing programs have shown promise in potentially combatting a number of health conditions and reducing loneliness (23), while leveraging off, and developing networks between existing services to develop more connected, supportive communities. We anticipate that this multi-pronged evaluation of *Connect Local* program will not only determine the effects on all program stakeholders, but also provide robust evidence to policy makers about the potential health and economic impact to enable optimisation of our health and social care systems. Further, this research will contribute to the development of a conceptual model to improve understanding of how program promoting social connection for older people can lead to improved health outcomes, wellbeing and reduced health service use.

## Data availability statement

The original contributions presented in the study are included in the article/supplementary material, further inquiries can be directed to the corresponding author.

## Author contributions

RO: Conceptualization, Methodology, Project administration, Writing – original draft, Writing – review & editing, Funding acquisition. ER: Methodology, Writing – original draft, Writing – review & editing. KR: Writing – review & editing, Methodology. SA: Methodology, Writing – review & editing, Writing – original draft. DF: Writing – review & editing, Funding acquisition, Methodology. KF: Writing – review & editing, Funding acquisition, Methodology. AY: Writing – review & editing, Methodology. ML: Writing – review & editing, Methodology. LE: Methodology, Writing – review & editing, Writing – original draft. JL: Conceptualization, Methodology, Writing – original draft, Writing – review & editing, Funding acquisition.
